# Towards the Configuration of a Photoelectrocatalytic Reactor: Part 1—Determination of Photoelectrode Geometry and Optical Thickness by a Numerical Approach

**DOI:** 10.3390/nano12142385

**Published:** 2022-07-12

**Authors:** Daniel Borrás-Jiménez, Wilber Silva-López, César Nieto-Londoño

**Affiliations:** 1 Grupo de Investigación en Óptica y Espectroscopía, Universidad Pontificia Bolivariana, Medellín 050031, Colombia; daniel.borras@upb.edu.co; 2 Grupo de Investigación en Energía y Termodinámica, Universidad Pontificia Bolivariana, Medellín 050031, Colombia; cesar.nieto@upb.edu.co

**Keywords:** photoelectrocatalytic reactor design, *TiO_2_* nanotube photocatalyst, tertiary wastewater treatments, computational fluid dynamics, mass transfer, radiation absorption, textile dye degradation

## Abstract

Photoelectrocatalysis has been highlighted as a tertiary wastewater treatment in the textile industry due to its high dye mineralisation capacity. However, design improvements are necessary to overcome photo-reactors limitations. The present work proposes a preliminary configuration of a photoelectrocatalytic reactor to degrade Reactive Red 239 (RR239) textile dye, using computational fluid dynamics (CFD) to analyse the mass transfer rate, radiation intensity loss (RIL), and its effect on kinetics degradation, over a photoelectrode based on a $TiO_{2}$ nanotube. A study to increase the space-time yield (STY) was carried out through mass transfer rate and kinetic analysis, varying the optical thickness (δ) between the radiation entrance and the photocatalytic surface, photoelectrode geometry, inlet flow rate, and the surface radiation intensity. The RIL was determined using a 1D Beer–Lambert-based model, and an extinction coefficient experimentally determined by UV-Vis spectroscopy. The results show that in RR239 solutions below concentrations of 6 mg/L, a woven mesh photoelectrode and an optimal optical thickness δ of 1 cm is enough to keep the RIL below 15% and maximise the mass transfer and the STY in around 110 g/m${}^{3}$-day.

## 1. Introduction

More than ten thousand different types of pigments and dyes are used by industries, such as textiles, paper, cosmetics, pharmaceuticals, among others, [1]. In particular, the textile industry discharges to the environment around 54% [2] of the total emissions in this regard, generating a huge amount of wastewater of approximately 125–200 L of water per kilogram of processed product [3]. Additionally, the percentages of fixation of dyes in textile fibres usually are between 50% and 98%; for example, reactive type dyes (which stand out due to the high demand worldwide [4]) have a percentage of fixation between 50% and 80% [5]. This low fixing capacity generates a high persistence of them in the wastewater. Moreover, the accumulation of dyes alters the biological activity of aquatic plants or algae [6], and some dyes have been shown to have high toxicity, carcinogenicity and mutagenicity [7,8,9]. Furthermore, due to the higher demand for water in textile industries and the high persistence of dyes, it is challenging to meet the Sustainable Development Goals (SDGs) regarding wastewater sanitation and the disposal of discharges.

Conventional wastewater treatment technologies consist mainly of physicochemical processes accompanied by a secondary biological degradation process. However, in these methods, the dye is not completely degraded due to the low biodegradability index [5,10], generating the need of tertiary treatment for the complete degradation of the dyes. The advance in tertiary wastewater treatment technologies points toward the study and development of more efficient oxidation processes, such as the so-called advanced oxidation processes (AOPs), which are based on the generation of highly oxidizing species such as hydroxyl radical (OH) [11]. Among the AOPs, there are technologies such as ozonation, Fenton-based processes, homogeneous and heterogeneous photocatalysis, among others. The last one is a promising technology due to its higher degradation capacity for recalcitrant contaminants, no subsequent separation process necessity, no secondary-pollution production and environmentally friendly designs [12]. It consists of using a photoactive catalyst (photocatalyst), a semiconductor that, when absorbing a UV/Vis photon, generates an electron/hole pair ($e^{-}/h^{+}$), where the $h^{+}$ can produce the radical OH by water oxidation. However, some semiconductors present a high rate of $e^{-}/h^{+}$ recombination; an annihilation of $e^{-}/h^{+}$ pair in the photocatalytic systems decreases the amount of OH radicals produced. Therefore, this technology’s advancement is limited to low degradation efficiencies [13]. The recombination rate can be decreased by applying an external polarisation potential, a technology called photoelectrocatalysis (PEC).

The PEC consists of the conformation of an electrochemical cell by integrating the photocatalyst into an anode (photoelectrode) and has received significant attention in the degradation of persistent pollutants due to the better use of the $e^{-}/h^{+}$ pair by decreasing recombination and maximising the production of the OH radical [14], making it an efficient and promising AOP for wastewater treatment [11], especially in textile dye degradation [15]. However, its development depends mainly on two factors: (i) the semiconductor material and (ii) the design of the electrodes/photoreactor [16].

Concerning material, there are many semiconductors with potential uses in photoelectrochemical cells, $TiO_{2}$, ZnO, $W_{2}O_{3}$ [13] and others. However, its applications are limited by the OH radical production kinetic, requiring a high production of radicals per second, and therefore low $e^{-}/h^{+}$ rate recombination. Although they have an acceptable properties for photocatalysis, developing new materials is necessary to maximise the OH radicals produced. Xiang et al. [17] experimentally determined a hydroxyl radical production rate of several photocatalysts, with the $TiO_{2}$ Degussa P25 and $TiO_{2}$ anatase phase being the most efficient. $TiO_{2}$ presents excellent quantum yield, high capacity for oxidation resistance, long-term stability, low preparation cost and low toxicity [18].

On the other hand, a new family of nanomaterials based on semiconductors has been established and used for photoelectrocatalysis systems. Compared to the conventional materials, nanostructured $TiO_{2}$, such as tubes, wires, fibres, and dots, among others, exhibits high photoelectric efficiency and photocatalytic activity for photodecomposition, due to its high surface area and changes in its electronic structure [19]. Among them, materials with a tubular structure have been considered suitable for achieving a more significant effective surface area enhancement without increasing the geometric area [20]. Furthermore, the nanotube structure is easily synthesised, compared to others [21]. A tubular $TiO_{2}$ nanostructure can be easily synthesised by titanium anodizing, and parameters such as nanotube length and diameter can be controlled to improve the photocatalytic properties. Zhang et al. [22] studied the effects of $TiO_{2}$ nanotubes with different thicknesses, annealing temperatures, and applied bias potential on the photoelectrocatalytic degradation of salicylic acid. They observed that the nanotube length until 8 μm improves photocatalytic degradation because the larger surface area is good for photon absorption, and the light can easily penetrate and scatter in such regular nanostructures. Ferraz et al. [23] studied the degradation of azo textile dyes, such as Dispersed Red 1, Dispersed Red 13, and Dispersed Orange 1, using $TiO_{2}$ nanotubes in a titanium carrier, demonstrated the effectiveness of photoelectrode ($Ti/TiO_{2}$) and photoelectrocatalysis process for degradation of these dyes, achieving a reduction in total organic carbon (TOC) greater than 87%, and therefore a decrease in mutagenic and cytotoxic activity in final waters. Additionally, $TiO_{2}$ nanotubes can be modified to improve photocatalytic activity. Wang et al. [24] developed a photoelectrode based on a p-n heterojunction of $TiO_{2}$ nanotubes with $Cu_{2}O$, which increases the separation of photogenerated electrons-holes and improves the absorption of visible light; therefore, the $TiO_{2}$/$Cu_{2}O$ nanotube heterojunction exhibited a more effective photoconversion capability than single $TiO_{2}$ nanotubes.

In the electrodes/photoreactor design, Hanking et al. [25] showed that a photoelectrode with three-dimensional support, such as a perforated plate, helps to homogenise the distribution of the ionic current. This arrangement influences the potential electrical distribution and, consequently, the degradation performance. In addition, they found that the inclusion of perforations in the photoelectrode offers a potential solution for photo-reactors because it decreases the magnitude in the ion current paths and the non-homogeneity in current density distributions. Bao et al. [26] developed a mesh photoelectrode with $TiO_{2}$ nanotube arrangements. They showed that employing the mesh electrode increases radiation uptake efficiency and photocatalytic activity, compared to two-dimensional support, and the internal stresses inside the nanotubes decrease when the nanotubes grow in the three-dimensional configuration of the support.

In contrast, photoelectrocatalytic reactors may have mass transfer limitations depending on the mass diffusivity of the contaminant, as demonstrated by Duran et al. [27] in their study of benzoic acid degradation by computational fluid dynamics (CFD) with experimental validation. Suhadolnik et al. [28] performed a laboratory-scale design, supported on CFD simulations, of a tubular photoelectrocatalytic reactor to degrade Reactive Red 106 by using a photoelectrode of $TiO_{2}$ nanotubes on two-dimensional sheets of titanium, concluding that the system is strongly limited by the low mass transfer rate of the dye. Jaramillo-Gutierrez et al. [29] proposed a tubular electrochemical photo-reactor of expanded mesh electrodes to increase the mass transfer rate. However, decisions in photoelectrode mesh and photoreactor sizing were not clear enough.

In these studies, there have been several approaches to preliminary design decisions, such as the inclusion of three-dimensional photoelectrode support and $TiO_{2}$ nanotubes as a promising photoelectrode material, as well as, CFD simulation has been a valuable tool to perform analysis in photoelectrocatalytic reactors with an acceptable precision between the phenomena and experimental data. However, the sizing of the photo-reactor and the photoelectrode support has not been studied enough; the possible losses of radiation intensity due to absorption by the pollutant have not been taken into account; and an analysis of the relationship between the mass transfer rate and dye degradation kinetics has not been studied enough, factors that can be decisive for the performance of a photoelectrocatalytic reactor. Therefore, for the PEC applied in dye degradation to have a technological advance at larger scales, research is needed to design this type of reactor to increase the mass transfer rate while maintaining a loss of low radiation intensity.

This article aims to propose a preliminary configuration of a photoelectrocatalytic reactor to degrade the textile dye Reactive Red 239, analyzing mass transfer rate, radiation intensity loss (RIL) and kinetics degradation. A computational fluid dynamics simulation approach was used to describe the phenomena of momentum transport (hydrodynamics), species transport and heterogeneous kinetics, and mathematical modelling based on the Beer–Lambert law for the RIL. First, an analysis of the effect of the distance between the entry of light and photoelectrode (optical thickness—δ) in the RIL was carried out through the Beer–Lambert law, using an extinction coefficient determined experimentally by UV-Vis spectroscopy. Then the effect of δ over two photoelectrode support geometries and their dimensions on the mass transfer rate by CFD were studied. To increase the mass transfer rate coefficient ($k_{m}$) without having high radiation intensity losses (<15%), an optimum δ and an electrode geometry were selected based on an objective function that relates both variables ($k_{m}$ and RIL). Finally, a geometric configuration of the photoelectrocatalytic reactor was proposed by performing an analysis of the influence of the flow regime concerning the mass transfer rate and degradation kinetics over the $TiO_{2}$ nanotube [28]. This work pretends to establish a basis for designing photoelectrocatalytic reactors for dye degradation.

## 2. Materials and Methods

The study of a photoelectrocatalytic reactor to degrade textile dyes using a numerical approach includes analysing mass transfer rate, radiation intensity loss (RIL) and kinetics degradation, among other variables that describe their influence on the overall reactor behaviour. In this regard, this section presents the approach followed to evaluate the photoelectrocatalytic reactor performance, considering first, a description of the coupled energy (including radiation transport model), mass, momentum and reaction governing equations solved using the CFD software ANSYS Fluent®. The governing equations, boundary conditions, and numerical solution strategy applied are presented. Then, the reactors’ model configuration is described and an objective function developed to relate the mass transfer rate coefficient with loss of radiation intensity is shown. Finally, it is explained the experimental method used to obtain the extinction coefficient through UV-Vis spectroscopy, which allows to study the RIL inside the photoreactor.

### 2.1. Governing Equations

#### 2.1.1. Momentum Transport Model

The photoelectrocatalytic reactor is studied under different inlet flow conditions, leading to the reactor’s operating in laminar and turbulent regimen conditions. Therefore, the Navier–Stokes equations with constant properties are used for the laminar regime cases, thus

(1) $$\rho \left(\upsilon \;\cdot \;▿\right)\upsilon =▿\;\cdot \;\left[-P+\mu \left(▿\;\cdot \;\upsilon \right)\right],$$

along with the continuity equation for an incompressible fluid,

(2) $$▿\;\cdot \;\left(\upsilon \right)=0.$$

In Equations (1) and (2), *u* refers to the flow velocity field, *P* is the fluid pressure, rho is the density, and mu is the dynamic viscosity.

For the turbulent flow regime modelling, the Reynolds-averaged Navier–Stokes equations (RANS) is assumed, in which the momentum transport equation takes the following form,

(3) $$\rho \left(\bar{\upsilon }\;\cdot \;▿\rho \bar{\upsilon }\right)=-▿\bar{P}+▿\;\cdot \;\left[\mu \left(▿\bar{\upsilon }+▿{\bar{\upsilon }}^{\left(t\right)}\right)+\tau^{T}\right],$$

being that $\tau^{T}$ is the Reynolds stress term, defined as

(4) $$\tau^{T}=\mu_{T}\left(▿\bar{\upsilon }+▿{\bar{\upsilon }}^{T}\right),$$

where the turbulence viscosity $\mu_{T}$, is expressed as,

(5) $$\mu_{T}=\frac{C_{\mu }\rho k^{2}}{\varepsilon }.$$

The standard κ−ε two-equation model allows evaluating the turbulent viscosity in Equation (5). In that expression, $C_{\mu }$ is an empirical constant whose default value is 0.09, κ is the turbulent kinetic energy, and ε is the turbulent energy dissipation rate. The wall presence affects the turbulence behaviour close to this region [30]. An enhancement wall treatment is used to improve the model accuracy. For that, a dimensionless velocity $u^{+}$ at the wall is smoothed by a damping function described by

(6) $$\upsilon^{+}=e^{\Gamma }\upsilon_{lam}^{+}+e^{1/\Gamma }\upsilon_{turb}^{+},$$

where the damping parameter Γ is defined as,

(7) $$\Gamma =-\frac{a{\left(y^{+}\right)}^{4}}{1+by^{+}},$$

and the dimensionless velocities for the laminar ($u_{lam}^{+}$) and the turbulent regime ($u_{turb}^{+}$) are, respectively,

(8) $$\upsilon_{lam}^{+}=y^{+},$$

(9) $$\upsilon_{turb}^{+}=\frac{1}{K}Ln\left(Ey^{+}\right).$$

The constants in Equations (7) to (9) are a=0.01, b=5, K=0.4187, and E=9.793. It should be noted that this wall treatment approach must be ensured that $y^{+}\approx 1$.

#### 2.1.2. Species Transport Model

A convection–diffusion model is used to evaluate the dye transport. The conservation equation is described by

(10) $$0=-▿N_{i}\pm R_{i},$$

where $R_{i}$ is the net rate consumption of the dye. The dye flux density $N_{i}$ is described with a diffusive term using Fick’s equation and with a convective term related to the velocity field calculated upon the momentum transport model (see Equations (1) to (3)), as follows

(11) $$N_{i}=-\rho D_{i}▿Y_{i}+\rho Y_{i}\upsilon ,$$

where ρ is the water-RR239 mixture density and the mass diffusivity $D_{i}$ of RR239 in water for the laminar regime. For its evaluation, an expression used by Suhadolnik et al. [28], expressed as

(12) $$D_{i}=\frac{1.7x10^{-7}T}{M^{0.41}\mu },$$

where *M* is the molecular weight of the dye (1136.32 kg/kmol), *T* is the mixture temperature (300 K) and μ is the dynamic viscosity of the water-RR239 mixture. Therefore, the density and viscosity properties of the mixture are closer to the values for the water (i.e., ρ=998 kg/m${}^{3}$ and μ=0.001 Pa.s), due to the lower RR239 concentrations. With these values, the RR239 mass diffusivity is $2.79\times 10^{-10}$ m${}^{2}$/s.

Regarding the turbulent regime, a turbulent diffusion term is added to the dye flux, as follows,

(13) $$N_{i}=-\left(\rho D_{i}+\frac{\mu T}{Sc_{T}}\right)▿Y_{i}+\rho Y_{i}\upsilon ,$$

In the same way as in the momentum transport model, in the turbulent species transport model, an enhancement wall treatment is used, in which the dimensionless mass fraction ($Y^{+}$) in the vicinity of the wall is defined as

(14) $$Y^{+}=e^{\Gamma_{S}}Y_{lam}^{+}+e^{\frac{1}{\Gamma_{S}}}Y_{turb}^{+},$$

where the parameter $\Gamma_{s}$ is evaluated as,

(15) $$\Gamma_{S}=-\frac{a{\left(Scy^{+}\right)}^{4}}{1+bSc^{3}y^{+}},$$

and the dimensionless mass fraction in the laminar $Y_{lam}^{+}$ and the turbulent $Y_{turb}^{+}$ regimes are, respectively,

(16) $$Y_{lam}^{+}=Scy^{+},$$

(17) $$Y_{turb}^{+}=Sc_{T}\left[\frac{1}{K}Ln\left(Ey^{+}\right)+Pc\right],$$

with the parameter Pc evaluated with the following expression,

(18) $$Pc=9.24\left[{\left(\frac{Sc}{Sc_{T}}\right)}^{\frac{3}{4}}-1\right]\left[1+0.28e^{-\frac{0.007Sc}{Sc_{T}}}\right],$$

where Sc is the molecular Schmidt number defined as

(19) $$Sc=\frac{\mu }{\rho D_{i}},$$

and $Sc_{T}$ is the turbulent Schmidt number. This number is calculated following an analogy between mass and heat transfer, with the heat and mass diffusivities equivalent ($\alpha_{t}=D_{\left(i,T\right)}$), and, consequently, the Prandtl and Schmidt numbers as well. In this way, the Kays–Crawford model is used to describe the turbulent Schmidt number, as,

(20) $$Sc_{T}=\left(\frac{1}{2Sc_{T_{\infty }}}+\frac{0.3}{\sqrt{Sc_{T_{\infty }}}}\frac{\mu_{T}}{\rho D_{i}}-{\left(0.3\frac{\mu_{T}}{\rho D_{i}}\right)}^{2}\left(1-exp\left(-\frac{\rho D_{i}}{0.3\mu_{T}\sqrt{Sc_{T_{\infty }}}}\right)\right)\right),$$

with a value of 0.85 for $Sc_{T_{\infty }}$ [29,31]. An user defined function (UDF) (see Appendix A.1) is used to include Equation (20) in the turbulent species transport model. Finally, the turbulent viscosity $\mu_{T}$ is obtained from the turbulent model described by Equation (5).

#### 2.1.3. Mass Transfer Coefficient

For the laminar regime, it is assumed that the RR239 transport towards the photoanode vicinity only occurs by diffusion to calculate the mass transfer coefficient $k_{m}$. This approach relies upon the low flow velocities in this area. Fick’s law approach is then used to calculate the flux density of the dye towards the photoanode by using the results of the mass fraction field of RR239, as

(21) $$J_{i,w}=-\rho D_{i}▿Y_{i}=-k_{m}\rho \left(Y_{\infty }-Y_{w}\right),$$

where $J_{i,w}$ is the dye diffusive flux density of the dye towards the photoanode, $▿Y_{i}$ is the dye mass fraction gradient in the vicinity of the photoanode, $Y_{\infty }$ is the mass fraction within the fluid taken as the input fraction, and $Y_{w}$ is the mass fraction at the photoanode surface.

On the other hand, for the turbulent regime, the dye flux towards the photoanode is calculated considering the following wall treatment function,

(22) $$J_{i,w}=\frac{\left(Y_{w}-Y_{p}\right)\rho C_{\mu }^{1/4}k_{p}^{1/2}}{e^{\Gamma }Y_{lam}^{+}+e^{1/\Gamma }Y_{turb}^{+}}=-k_{m}\rho \left(Y_{\infty }-Y_{w}\right).$$

For both laminar and turbulent regimes, $k_{m}$ is calculated using a UDF once the results of the mass fraction field are obtained. The algorithm for calculating $k_{m}$ in both regimes is shown in Appendix A.2.

#### 2.1.4. Radiation Model

The steady-state general radiation transport equation (RTE) relates to the change in the intensity radiation in space, with the radiation gains or losses due to the scattering or absorption of the medium. For a given wavelength, the RTE is defined as

(23) $$▿\;\cdot \;\left(I_{\Omega ,\lambda }\Omega \right)=W_{\Omega ,\lambda }^{in-s}-W_{\Omega ,\lambda }^{ab}-W_{\Omega ,\lambda }^{out-s},$$

where $I_{\Omega ,\lambda }$ is the radiation intensity with direction Ω and wavelength λ, $W_{\Omega ,\lambda }^{in-s}$ is the scattering radiation gain, $W_{\Omega ,\lambda }^{ab}$ is the loss of radiation by absorption of the medium, and $W_{\Omega ,\lambda }^{out-s}$ is the scattering radiation loss.

The water-RR239 domain is treated as a continuous medium without suspended particles; therefore, in the RTE model, it is possible to neglect dispersion terms [32], leaving only the medium absorption contribution. The radiation transport equation, assuming a one-dimension approach, simplifies to the Beer–Lambert law, defined as

(24) $$\frac{dI_{s,\lambda }}{ds}=-\kappa_{\lambda }I_{s,\lambda },$$

where $I_{s,\lambda }$ is the radiation intensity in the *s* direction for a specific wavelength, λ is the wavelength of the incident radiation and $\kappa_{\lambda }$ is the light absorption or extinction coefficient. The $\kappa_{\lambda }$ coefficient is a constant related to the capacity of a chemical species to absorb light at a given wavelength; therefore, it depends mainly on the dye concentration in the aqueous medium. It is calculated experimentally with UV-Vis spectroscopy, as will be mentioned in Section 2.5. Based on Equation (24), an expression is proposed to calculate an approximation of the radiation intensity loss in a fast and simple way.

#### 2.1.5. Kinetic Model

Photocatalyst film’s surface reactions in a photoelectrocatalytic system are complex electrochemical and homogeneous phenomena reactions. However, the global reaction can be simplified using empirical equations, accounting for the contribution of the most significant variables, such as the surface intensity of radiation, the concentration of the dye, and the voltage effect,

$$RR239_{dye}+Photocatalyst+UV\overset{current}{\to }Products.$$

For this study, the following superficial photoelectrocatalytic degradation kinetics for $TiO_{2}$ nanotubes is used,

(25) $$r_{RR239}=\left(I_{UV}+I_{0}\right)I_{c}k_{reac}\rho_{c}C_{dye},$$

where the effects of surface radiation intensity $I_{UV}$ in W/m${}^{2}$ and electric current $I_{c}$ in Ampere (A) are included; additionally, $I_{0}$ represents the kinetic decomposition under conditions without illumination in W/m${}^{2}$, $k_{reac}$ a kinetic constant, $\rho_{c}$ the surface area per unit length (m${}^{2}$/m) and $C_{dye}$ the dye surface concentration. Equation (25) was obtained for a microreactor [28] under conditions that ensured the analysis was performed with no significant mass transfer effects, and the surface kinetics completely limited the overall reaction. The surface reaction kinetic was implemented as a UDF (see Appendix A.3).

### 2.2. Boundary Conditions

**Figure 1:**
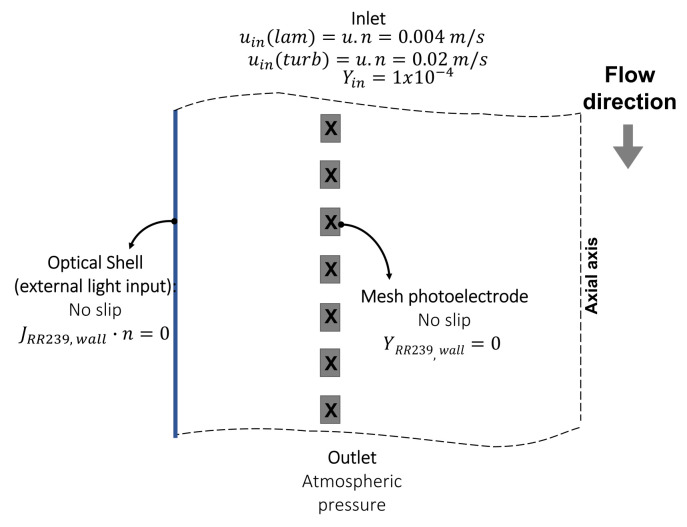
Boundary conditions for studying the mass transfer flux rate.

The boundary conditions for studying the mass transfer flux rate during the photoanode selection and the photoreactor configuration study are shown in Figure 1. As a simulation strategy, a rotational periodic geometry for the mesh electrode was used (88, 8° of all tubular geometry). For the cathode area in the proposed photoreactor configuration study, it was taken into account the previously mentioned area ratio (presented in Section 2.4.2) and a photoanode area of 82.5 cm${}^{2}$ (value taken in 8 cm length of the developed zone) in the periodic cell.

The boundary conditions for the momentum and species transport model are as follows:

1. At the entrance, the flow velocity is specified depending on the hydrodynamic regime and the mass fraction of the dye. In the case of the turbulent regime, the hydraulic diameter is defined according to the geometry variation, and the flow turbulence intensity is set equal to 5%.
2. For all the solid surfaces, the non-slip condition is set. Additionally, a zero mass fraction of RR239 was set at the photoanode, while for the rest of the wall surfaces, a zero diffusive flow rate of the RR39 normal to the frontier is defined.
3. Periodic boundaries without pressure drop for the lateral faces.
4. An exit boundary condition at atmospheric pressure.

The same boundary conditions are used for the photoreactor configuration study, except for the photoanode surface, where the surface reaction kinetics is implemented as a UDF as mentioned above. A constant radiation intensity over the entire photoanode surface is assumed in the reaction kinetic. For this, an average solar radiation of 4.5 kWh/m${}^{2}$ day (187.5 W/m${}^{2}$) was assumed for the city of Medellín-Colombia [33]; on this value, the 7% was calculated as an approximation of the UVA radiation intensity (13.2 W/m${}^{2}$). The latter value was taken as the radiation intensity on the optical shell of the photoreactor, and with the percentage of UVA-average radiation intensity loss ($RIL_{avg}^{UVA}$ calculated with Equation (32), the surface intensity radiation was determined on the photoanode as 11.6 W/m${}^{2}$.

### 2.3. Numerical Solution Method

The governing equations for the momentum and species transport were solved through the finite volume method implemented in the commercial CFD software ANSYS Fluent^®^. The SIMPLE coupling and second-order upwind discretisation schemes were used. Simulation convergence was achieved when residuals were lesser than $1\times 10^{-5}$ for each of the transport properties, and the standard deviation of the mass fraction at the outlet was stable. Additionally, in the photoreactor configuration study, it was also monitored that the RR239 averaged mass fraction over the entire photoanode surface was stable. Finally, the $y^{+}$ value was monitored throughout the iterative process regarding the turbulent regime. This value is set to be approximately 1 or lesser, ensuring the accuracy of the wall treatment. In Section 3.1, independence mesh and converge studies are presented to assess the numerical solution applied for the photoreactor study.

### 2.4. Computational Domains

#### 2.4.1. Photoanode Mass Transfer Velocity

A photoreactor with 9.7 cm diameter is used to investigate the effect of the photoanode geometry and its position inside the reactor over the mass transfer velocity and RIL. The mesh photoanode is positioned at a distance δ from the external surface of the tubular photoreactor. The cathode was not taken into account because the study focuses only on the effect of the photoanode.

**Figure 2:**
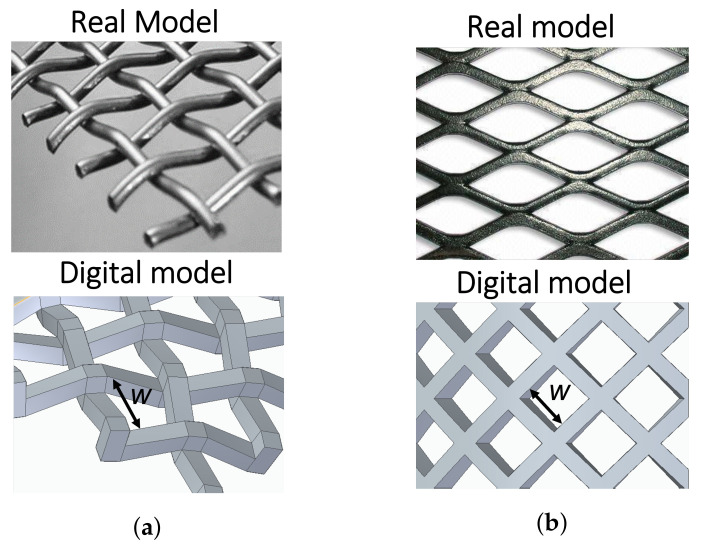
Real and digital model of (**a**) woven mesh electrode (WME), and (**b**) expanded mesh electrode (EME).

The effect of the optical thickness δ and the dimensions of the mesh electrode on the mass transfer rate are evaluated using a CFD solution approach. Two geometric photoanode configurations are used, i.e., woven mesh electrode (WME) and expanded mesh electrode (EME), as depicted in Figure 2. The geometry models are made in such a way as to facilitate the computational meshing procedure associated with the numerical solution method used.

At first, the surface area of both photoanode mesh configurations (WME and EME) is varied, changing the mesh aperture (*w*) for values equal to 0.3, 0.4, and 0.6 cm, keeping constant the characteristic optical thickness δ to 0.4 cm, and a constant flow velocity of 0.004 m/s (which give rise to laminar flow conditions, with Reynolds number in the range between 135 and 180). It allows establishing the influence on the mass transfer coefficient $k_{m}$ for that set of configuration parameters. Then, those photoanode mesh configurations, classified according to the mesh opening *w*, generating the highest mass transfer rate are used to evaluate the impact on $k_{m}$, varying the optical thickness δ 0.1, 0.4 and 1 cm and the flow regime. For the latter, a constant flow velocity of 0.004 m/s (174<Re<196) leads to a laminar regime within the photoanode. Further, the flow velocity is raised to 0.02 m/s (870<Re<980) to account for the turbulence effect on the mass transfer coefficient.

**Figure 3:**
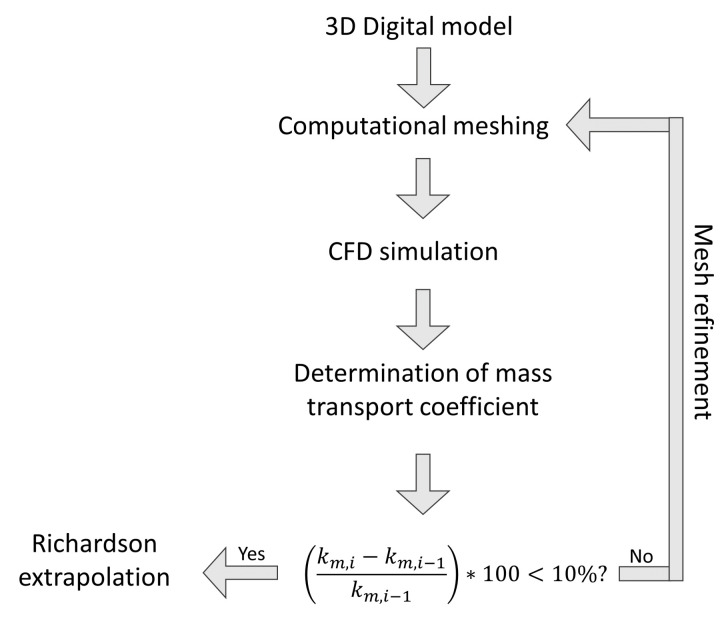
Computational procedure to study the mass transfer rate.

The general procedure used for the computational study is shown in Figure 3. First, a three-dimensional geometry model is created following the specific dimensions *w* and δ; then, an unstructured computational meshing is defined with tetrahedral elements by controlling the element’s size in the electrode surface and optical shell.

Subsequently, the solution of the governing equations (see Section 2.1, Section 2.2, Section 2.3, Section 2.4 and Section 2.5) is carried out. The mass transfer rate coefficient is calculated considering the simulation results using a user defined function (UDF) defined in Appendix A.2 as mentioned above. Once $k_{m}$ is calculated, the computational mesh is refined by modifying the element size on the electrode and optical shell surface until the difference in $k_{m}$ is less than 10% compared to the $k_{m}$ obtained with the previous mesh size. Finally, the calculation of the $k_{m}$ with an infinite mesh is made using Richardson’s extrapolation, taking into account the methodology reported in [34].

#### 2.4.2. Photoreactor Evaluation

The results of the mass transfer rate coefficient as a function of δ, the flow regime, and the radiation intensity loss analysis are used to obtain an objective function for finding the δ that maximises $k_{m}$ and minimises RIL, expressed as,

(26) $$O.F.=\left(\frac{k_{m}}{k_{m|max}}\right)\left(1-RIL\right).$$

The electrode geometry with the highest mass transfer rate is taken into account. The $k_{m}$ data as a function of δ is normalised regarding the maximum $k_{m}$ in an interval between 0 to 1 cm. An expression of ($k_{m}/k_{m|max}$) as a function of δ is obtained to implement it in the objective function Equation (26), for both laminar and turbulent flows. The RIL is defined, bearing in mind the expression defined in Section 2.5.

**Figure 4:**
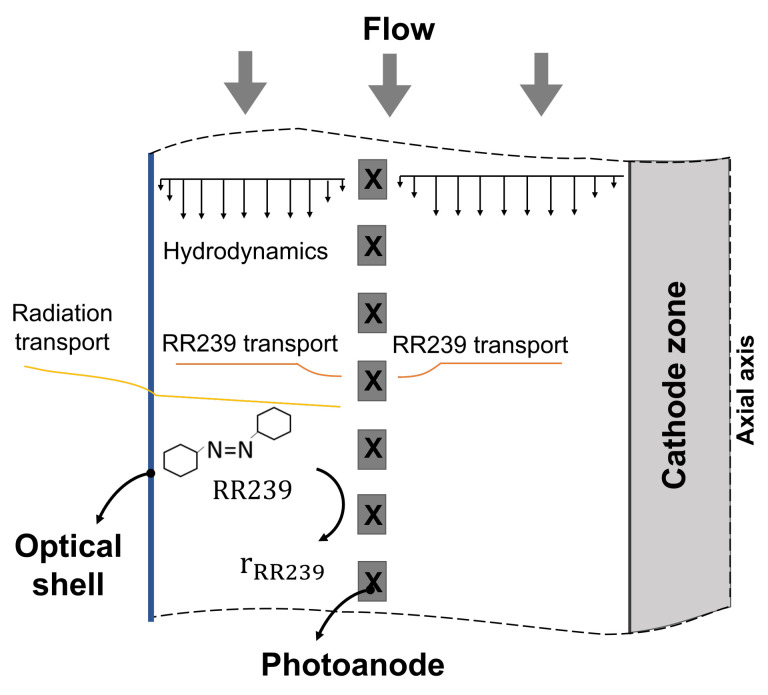
Photoreactor simplified computational model.

Once the distance δ is selected, a CFD study of a tubular photoelectrocatalytic reactor is carried out using the selected photoanode configuration, as shown in Figure 4. A 2.7 $A_{photoanode}/A_{cathode}$ ratio is used to calculate the cathode area and the empirical photoelectrocatalytic kinetic model (presented in Section 2.1.5) is used for dye consumption at the photoanode surface. Both the area ratio and the kinetics were obtained from [28].

Next, the flow velocity is varied, and the space–time yield factor (STY) is calculated only in the developed flow zone to maximise it. This factor was proposed by [35] and relates the mass of degraded dye per unit of time and reactor volume, as follows:

(27) $$STY=\frac{m}{V_{reac}t}=\frac{\dot{m}}{V_{reac}}.$$

Furthermore, the surface radiation intensity in the kinetic reaction is varied at a constant flow rate selected after the analysis explained above. Finally, the external effectiveness factor ($E_{ex}$) is calculated as

(28) $$E_{ex}=\frac{k_{eff}}{k_{reaction}}.$$

This factor determines if the global process is limited by the mass transfer rate (i.e., when $E_{ex}$ is close to 0) or by the surface kinetics (i.e., $E_{ex}\approx 1$), which depends on the effective reaction rate ($k_{eff})$ defined as,

(29) $$k_{eff}=\frac{k_{reaction}k_{m}}{k_{reaction}+k_{m}}.$$

In Equation (27), *m* is the mass of dye degraded in a specific reactor volume ($V_{reactor}$) at a time *t*, and $k_{reaction}$ is the first-order reaction rate.

### 2.5. Radiation Intensity Losses Evaluation

**Figure 5:**
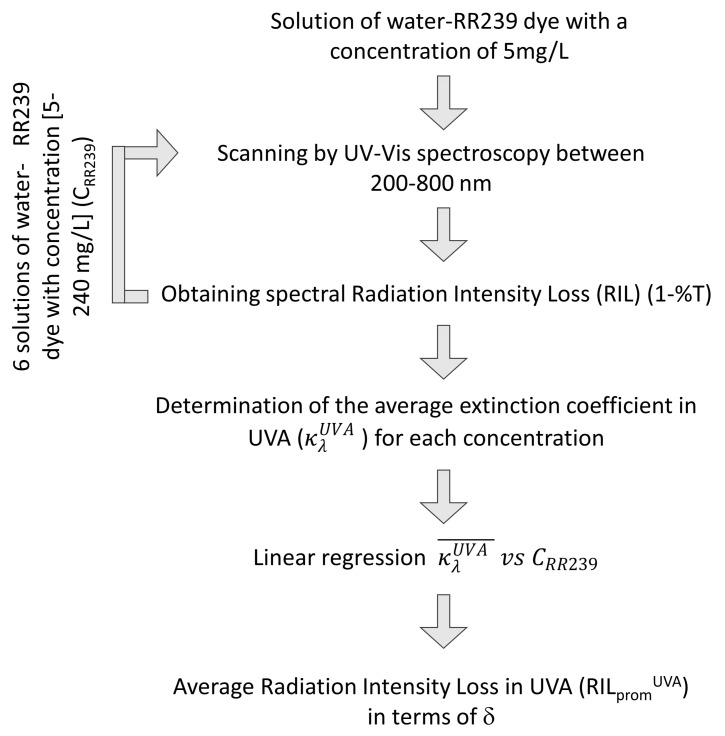
Procedure followed for determining the RIL as a function of δ.

The radiation intensity losses are characterised following the procedure described in Figure 5. First, the transmittance spectrum (%T) for seven water-RR239 dye solutions, with a concentration between 5 and 240 mg/L, is measured by UV-Vis spectroscopy (*Termo Scientific Genesys 6*, 1 cm cell, 200–700 nm spectrum). Then, based on the %T values, the RIL for the water-dye concentrations range is estimated.

The spectral extinction coefficient ($\kappa_{\lambda }$) is determined following the Beer–Lambert radiation model for each concentration of RR239 using the obtained RIL and the spectrophotometer cell thickness as the characteristic optical distance (i.e., 1 cm). The calculated extinction coefficient presents no variation for different characteristic optical distances since it only depends on the wavelength and the dye concentration. In this sense, using $\kappa_{\lambda }$, the RIL is calculated for δ equal to 0.1 and 0.4 cm, as follows:

(30) $$RIL_{\lambda ,C_{dye},\delta }=1-e^{\kappa_{\lambda ,C_{dye}}\delta }.$$

It is worth mentioning that Equation (30) is applied for each wavelength and specific dye concentration, allowing the evaluation of the RIL between 200–700 nm, different RR239 concentrations and δ (i.e., 0.1, 0.4 and 1 cm). In this way, a weighted average extinction coefficient in the UVA wavelength spectrum for each concentration value of RR239 ($\kappa_{\lambda }^{UVA}$, 315–380 nm), is obtained as

(31) $$\bar{\kappa_{\lambda }^{UVA}}=\frac{\int \kappa_{\lambda }d\lambda }{\int d\lambda }.$$

The above result permits to obtain a UVA average RIL, in terms of δ and RR239 concentration, using the Beer–Lambert model by applying a linear regression for $\kappa_{\lambda }^{UVA}$, thus,

(32) $$RIL_{aveg}^{UVA}=1-e^{aC_{dye}\delta },$$

where *a* is a constant determined experimentally. The UVA average is taken due to the $TiO_{2}$ absorption spectrum, one of the most used photocatalysts shown to be effective in dye degradation [23]. 32 is used to determine the surface radiation intensity on the photoanode, which will be explained in Section 2.2.

## 3. Results and Discussion

### 3.1. Convergence and Mesh Independence Study

**Figure 6:**
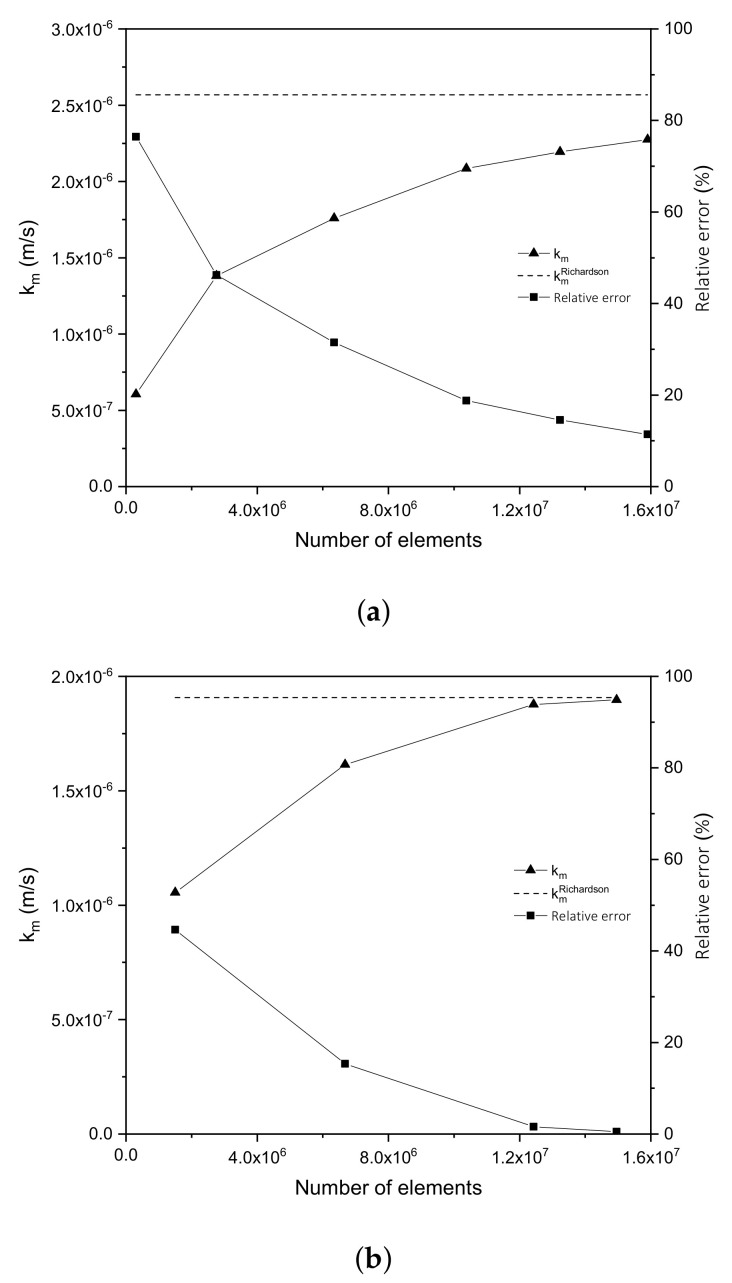
Convergence results for $k_{m}$ in laminar flow with *w* equal to 0.6 cm and δ equal to 1 cm for (**a**) WME and (**b**) EME.

In order to validate the solution methods presented in Section 2, a mesh independence study was carried out. Figure 6 shows the results for a reference case study with a fixed value for the electrode mesh aperture *w* equal to 0.6 cm and a distance δ of 1 cm. The mesh independence study was developed for both electrode geometries described in Section 2.4 under laminar and turbulent flow conditions, but only the laminar regime result is shown. It is important to mention that the mesh independence study was carried out only by varying the size of the computational mesh. It is observed from the results for the WME (Figure 6a) and EME (Figure 6b) electrodes that meshes within 13 and 15 million elements present a relative error lesser than 10% in both cases (square symbol). Additionally, a convergent behaviour (triangle symbol) towards the value of the mass transfer coefficient obtained by Richardson extrapolation (dotted line) is observed; a similar behaviour was previously observed for other surface coefficients, such as the convective heat transfer [34].

For the Richardson extrapolation, the last $k_{m}$ values obtained are analysed, ensuring a ratio between the fine and coarse mesh size (coarse mesh size / fine mesh size) greater than 1.2 to increase the extrapolation precision. The mesh independence studies present a similar behaviour for both configurations, as shown in Figure 6, with a relative error of less than 12% when the mesh size is approximately 15 million elements. Therefore, the selected mesh element size in the photoelectrode surface and the optical shell is 3 × 10 ${}^{-4}$ and 4 × 10${}^{-4}$ m, respectively.

### 3.2. Ril Characterisation

**Figure 7:**
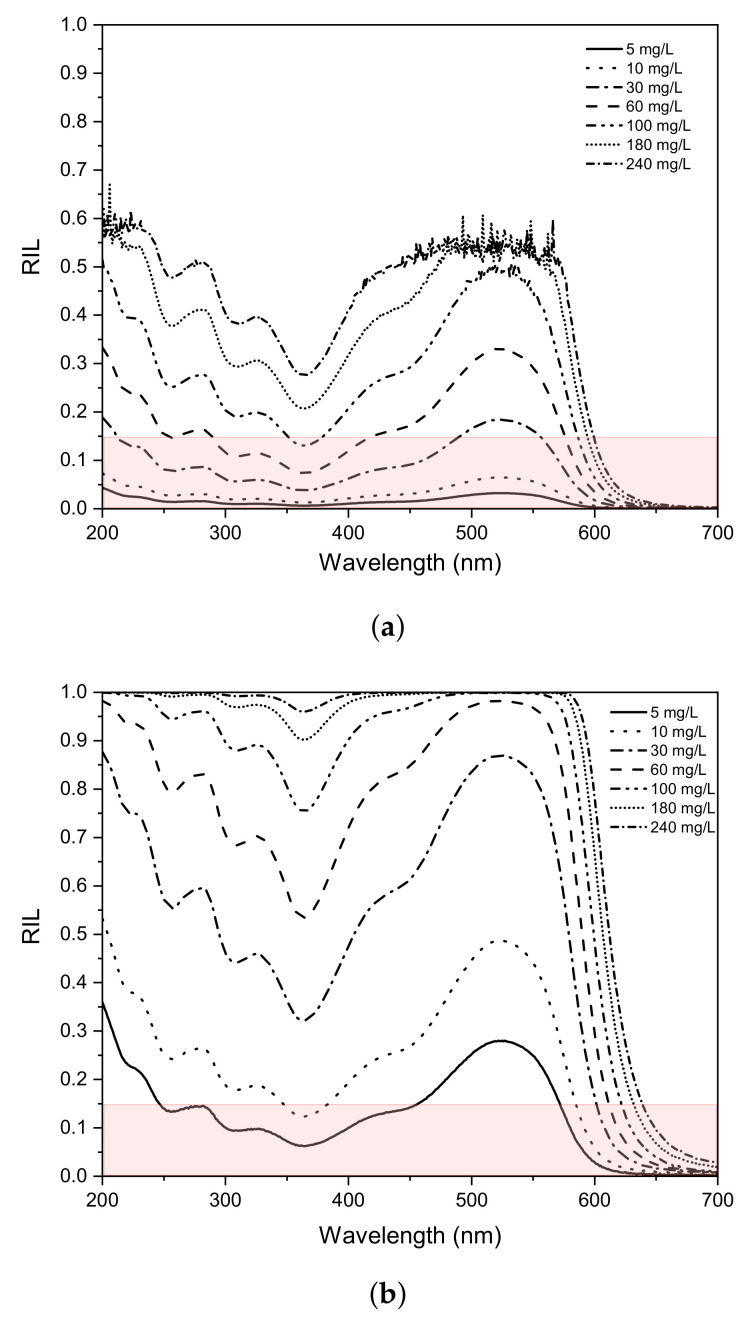
RIL spectra with different RR239 concentration for δ equal to 0.1 cm (**a**) and 1 cm (**b**).

An assessment of the radiation loss as a function of concentration and electrode depth within the reactor is performed to determine the amount of effective radiation reaching the catalyst surface following the procedure explained in Section 2.5. Figure 7 shows the RIL corresponding to the 200 and 700 nm wavelength range, with δ equal to 0.1 cm (Figure 7a) and 1 cm (Figure 7b), varying the RR239 concentration between 5 and 240 mg/L. It is observed that a more considerable value of δ, the RIL increases significantly. For δ equal to 0.1 cm, and all the wavelengths evaluated, the RIL remains less than 60%, while for a value of δ of 1 cm, 100% of the RIL is reached. This mainly occurs at high dye concentrations (>180 mg/L), as shown in Figure 7b; it indicates that no significant radiation would reach the electrode surface upper this distance and dye concentration.

In the spectra presented in Figure 7a,b, a strong dependence of RIL on RR239 concentration is observed, particularly in the visible spectrum region (λ > 380 nm), where the RIL is larger than the UV spectrum region, for any dye concentration. A minimum of RIL was found at approximately 365 nm in the UV spectrum region; this corresponds to a region presenting no electronic transition in the dye. This indicates that 365 nm could be the wavelength used in an artificial emission system where the photocatalyst absorbs radiation in the UV region.

From the results in Figure 7, it is possible to deduce the maximum theoretical RR239 concentration in which lower RIL is obtained. In the pink box, it is observed that by having RIL less than 15% with a δ of 0.1 cm, and if the dye concentration is greater than 30 mg/L, it is impossible to operate with a photocatalyst that absorbs in the visible spectrum region. However, if the δ is 1 cm, it is not possible to use in the visible spectrum for any RR239 concentration due to the significant value of RIL (>15%). Therefore, when a PEC reactor design is carried out for dye degradation, the dye concentration, the optical thickness δ, and the absorption spectrum of the photocatalyst must be taken into account to improve the energy efficiency and radiation absorption performance by the photocatalyst. It is essential to highlight that high dye concentrations could limit the application of the technology for photocatalysts that absorb in the visible spectrum; in these cases, a photocatalyst with absorption in the UV could be adequate.

In the vicinity of the photoelectrode, there is a lower dye concentration due to the surface reaction of degradation; therefore, it is possible that the radiation intensity loss is lower in this zone. However, in this work, an analysis is made by taking into account a constant dye concentration in the axial direction to approximate the maximum radiation loss reached in the photoreactor. To take into account the radiation intensity profile in the vicinity of the photoelectrode, the species transport model must be coupled with the radiation transport model. For the latter, more complex three-dimensional models must be used that capture the three-dimensional geometry of the photoelectrode, which is not part of the scope of this work.

**Figure 8:**
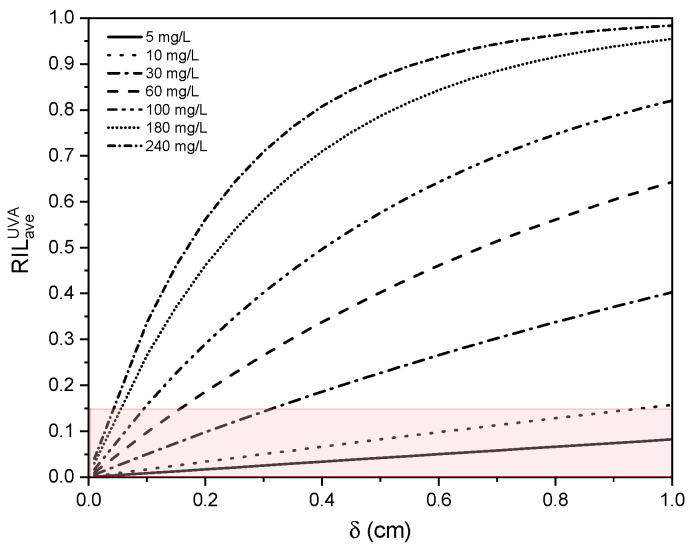
UVA-average RIL in terms of δ and RR239 concentration.

A UVA-average extinction coefficient function ($\kappa_{ave}^{UVA}$) to establish the best way to quantify the maximum radiation intensity that reaches the photoanode in the UV spectrum, was determined in terms of dye concentration, given as

(33) $$\kappa_{ave}^{UVA}=1.72\times 10^{-2}C_{RR239}$$

where $C_{RR239}$ is the dye concentration in mg/L. The analysis is performed in the UVA region, considering the average RIL because $TiO_{2}$ absorbs in this spectrum region. Considering the expression of the UVA-average extinction coefficient described by Equations (28) and (32), presented in Section 2.5, the behaviour of the average RIL in UVA in terms of δ and dye concentration was determined, as depicted in Figure 8. It is observed that the RIL increases significantly with larger values of δ for high concentrations of RR239 (>100 mg/L), presenting RIL values greater than 50% even in small values of δ (<0.4 cm).

### 3.3. Hydrodynamics Characterisation

The hydrodynamic behaviour is considered by analysing the velocity profiles to determine the best electrode location inside the photoreactor. It includes the effect of the electrode’s aperture width *w*, the distance between the entrance of the radiation and the photoanode surface δ and the geometry of the photoelectrode. The effect of δ (0.1, 0.4, and 1 cm) and *w* (0.3, 0.4, and 0.6 cm) was studied for both the WME and EME electrode geometry in laminar and turbulent regimes. The results are presented for WME, and for EME only with *w* equal to 0.6 cm. This is due to the fact that the WME geometry presented the best results in terms of mass transfer rate compared to EME, as well as EME (with *w* equal to 0.6 cm) compared to the other variations of *w* in the same electrode geometry; in addition, the results are presented in such a way to visualise the tendency and the analysis that it wants to explain. It should be noted that for the written analysis, all the results are taken into account.

**Figure 9:**
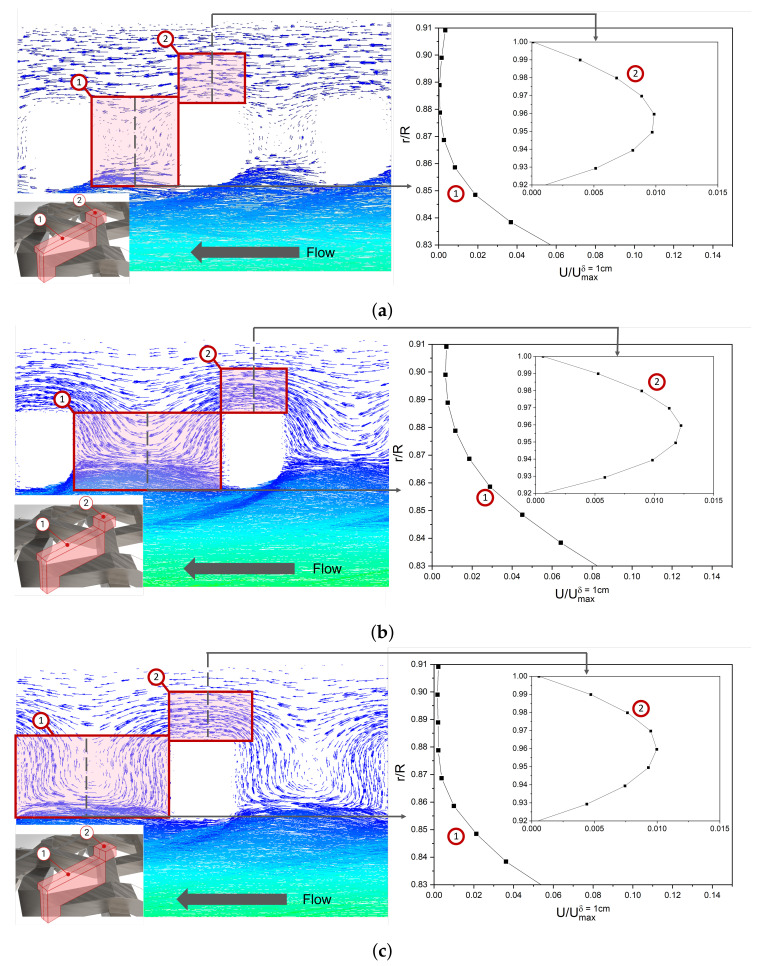
Velocity vectors and velocity magnitude profiles in laminar regime with constant δ (0.4 cm) and *w* equal to (**a**) 0.3 cm (**b**) 0.6 cm with WME and (**c**) 0.6 cm with EME.

Figure 9 shows the velocity vectors, obtained by CFD simulation, for the WME geometry with constant δ of 0.4 cm and *w* varying thus, 0.3 (Figure 9a) and 0.6 cm (Figure 9b), and EME with *w* equal to 0.6 cm (Figure 9c). It is found that an increase in *w* represents more significant fluid flow interaction in the mesh aperture for both WME and EME geometry. It is observed from Figure 9a,b that there is a higher velocity vector density in the mesh aperture (pink box 1) and mesh surface (pink box 2) when *w* increases, representing a greater velocity in these zones and a more interaction with the electrode surface. It can be observed in the velocity magnitude profile shown at right side of the Figure 9a,b, that the velocity magnitude in both zones is greater when *w* increase. Furthermore, when the WME geometry is used, more fluid element interaction is observed than with EME geometry, which is due to the fluid hydrodynamic patron which leads to a greater velocity in both zones, as shown in Figure 9b,c.

**Figure 10:**
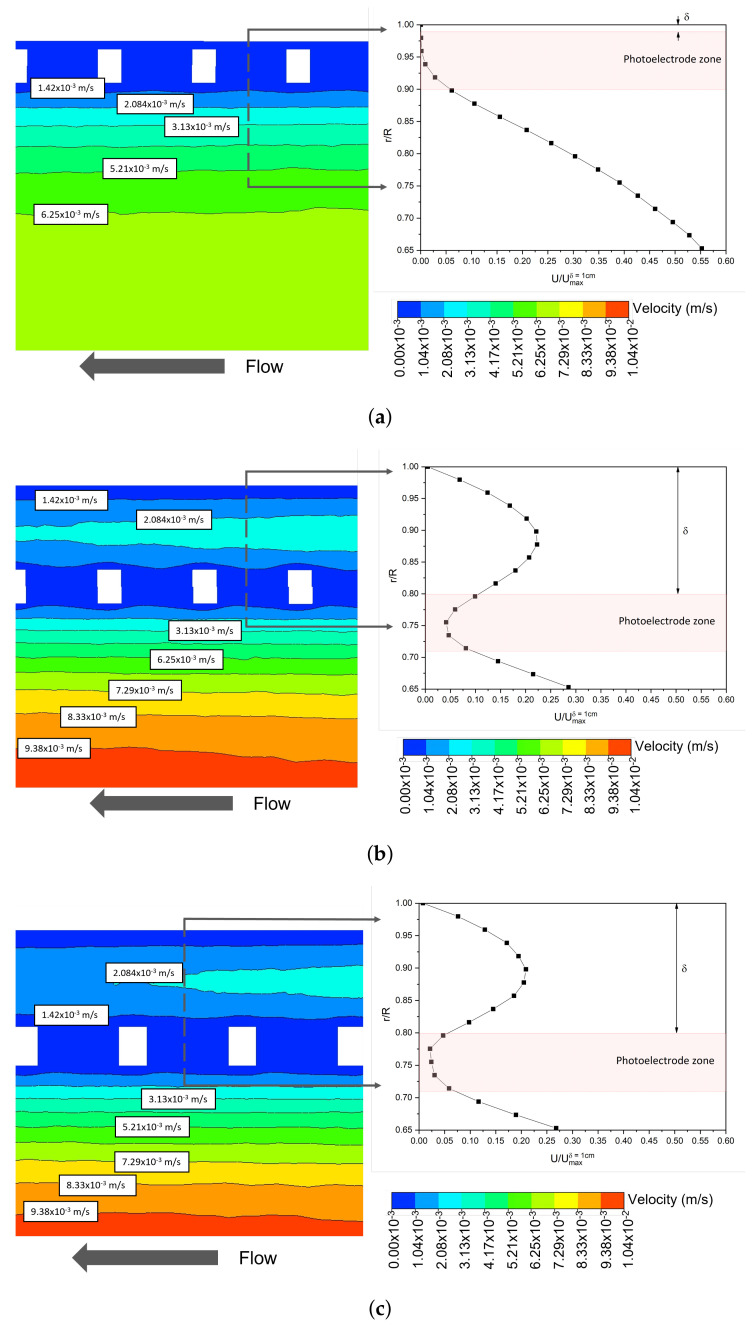
Contour plots and velocity magnitude profiles in laminar regime with *w* constant (0.6 cm) and δ equal to (**a**) 0.1 cm, (**b**) 1 cm with WME and (**c**) 1 cm with EME.

Figure 10 shows the velocity contour plots and velocity magnitude profiles for the WME geometry with δ of 0.1 (Figure 10a), 1 cm (Figure 10b), and the velocity contour for the EME geometry with δ of 1 cm in Figure 10c. The velocity profiles are normalised for the maximum velocity in the WME and δ of 1 cm ($U_{max}^{\left(\delta =1cm\right)}$). It is observed that for a value of δ equal to 0.1 cm, a stagnant zone is created with low velocities (<1.42 × 10${}^{-3}$ m/s, in dark blue colour) between the optical shell and photoelectrode (it was also observed for δ equal to 0.4 cm), which would not benefit the mass transfer velocity due to diffusion limitation. This tendency is observed in both electrode geometries under laminar and turbulent regimes. Therefore, with a δ = 1 cm, an increase in velocity is attainable, reducing the flow stagnation.

**Figure 11:**
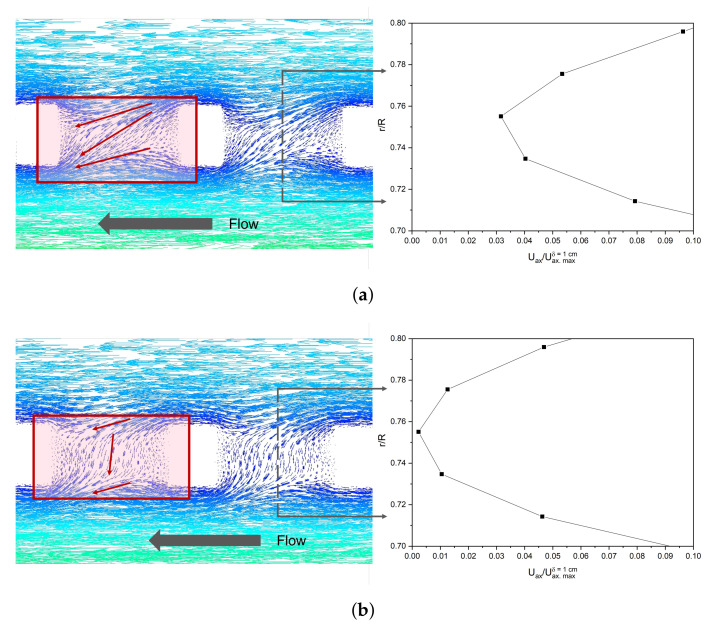
Velocity contour plots and axial velocity profiles in laminar regime with δ equal to 1 cm and *w* 0.6 cm for (**a**) WME and (**b**) EME.

It is important to emphasise that with the WME geometry, higher velocities are generated in the photoelectrode zone (pink box in the axial velocity profile of Figure 10) for δ of 1 cm, compared to EME. This could be due to higher levels of micromixing in the WME geometry as a consequence of the hydrodynamic profile shape in the vicinity of the electrode. Figure 11 shows the velocity vectors with δ equal to 1 cm and *w* of 0.6 cm, for WME (Figure 11a) and EME (Figure 11b). It is observed that in the mesh aperture, the hydrodynamic profile of WME is Z-shaped, and the EME is I-shaped, generating a higher axial fluid velocity in this area for the WME (three times greater than EME), as shown in the axial velocity profiles on the right side. In addition, the Z-shaped profile in the WME geometry generates a kind of shock towards the electrode surface, which could reduce the thickness of the boundary layer of the mass fraction of RR239.

### 3.4. Mass Transfer Coefficient

**Figure 12:**
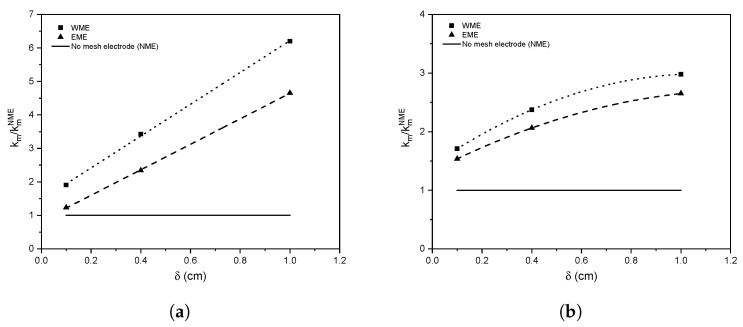
Mass transfer coefficient obtained with constant *w* (0.6 cm) and varying δ in (**a**) laminar and (**b**) turbulent regime.

Based on the species transport modelling, it was determined that increasing the value of *w* makes the mass transfer coefficient longer, which corresponds with the previously hydrodynamic characterisation analysis. This tendency was more evident in the WME geometry; however, a *w* of 0.6 cm was chosen in both geometries to perform the analysis varying δ. Figure 12 shows the relation of $k_{m}$ for a reactor with mesh electrode WME and EME, concerning the $k_{m}$ in the same reactor without a mesh electrode, calculated under the laminar regime (Figure 12a) and turbulent regime (Figure 12b) all in terms of δ. The reported values were obtained with the Richardson extrapolation.

There is a tendency to significantly increase the mass transfer rate by increasing the value of δ for both electrode geometries, a linear trend in the laminar regime and polynomial in the turbulent regime. This behaviour for the laminar and turbulent regimes is consistent with what was observed in other works [29,36]. It is noted that in both laminar and turbulent regimes, the WME geometry produces a greater mass transfer rate. This is due to a more significant fluid interaction as mentioned above in the hydrodynamic characterisation, increasing the micromixing in the vicinity of the electrode and the mass transfer rate, making the WME configuration an excellent geometry to increase the mass transfer rate in this type of system.

In the laminar regime, a mass transfer rate of six and four times superior is obtained with the WME and EME geometry, respectively, compared to the flat surface case. In the turbulent regime, the mass transfer rate is three times greater than no mesh electrode case. This behaviour is due to the chaotic nature of the turbulent regime, which causes higher levels in all directions even without having a turbulence generator, such as the mesh electrode. For this reason, the effect of the micromixing caused by the photoelectrode becomes more significant in the laminar regime.

### 3.5. Optimisation

An objective function was elaborated (see Equation (26)) to determine a suitable δ that increases the RR239 mass transfer rate with low radiation intensity loss. This objective function relates the RR239 transport with the mass transfer rate coefficient and the percentage of radiation transmittance for each value of δ.

**Figure 13:**
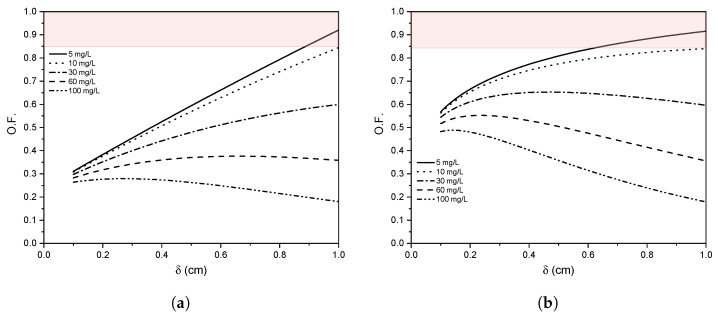
Objective function using WME in (**a**) laminar and (**b**) turbulent regime.

Figure 13a,b shows the objective function behaviour for laminar and turbulent flow using a WME configuration, respectively. The objective function for the laminar and turbulent flows shows a trend defined by the $k_{m}$ in both hydrodynamic regimes. It is possible to obtain objective function values close to 1 (high mass transfer rate and low RIL) for RR239 concentrations less than 10 mg/L in both laminar and turbulent regimes. However, when the concentration of RR239 is greater than 30 mg/L, there is a substantial limitation by the RIL when increasing δ. In this regard, it is observed that there is a maximum value of the objective function for δ equal to 0.8 and 0.4 cm for 30 mg /L in the laminar and turbulent regimes, respectively. However, when the RR239 concentrations are greater than 60 mg/L, it forces the use of smaller δ values due to the strong limitation by the RIL, which decreases the rate of mass transfer.

### 3.6. Photoelectrocatalytic Reactor Configuration Study

**Figure 14:**
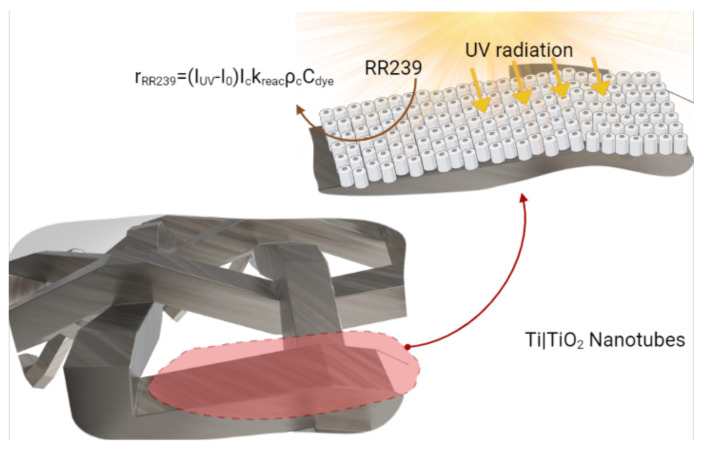
Superficial degradation reaction on titanium dioxide nanotubes.

The proposed photoelectrocatalytic reactor configuration consists of a woven mesh photoanode coated with titanium dioxide nanotubes, an efficient photocatalyst to degrade textile dyes. Over the photoanode surface, the degradation reaction of the RR239 dye is carried out, as shown in Figure 14. An empirical reaction kinetics was used on TiO${}_{2}$ nanotubes, in which the reaction mechanism is simplified by an equation that relates the main parameters that affect the dye degradation process; these are radiation term, current, and the surface dye concentration, as shown in Equation (25). A dye concentration of 6 mg/L is selected for a new PEC configuration study. Therefore, considering the optimisation analysis mentioned above, it is possible to select a δ of 1 cm to have a high mass transfer rate with low RIL.

**Figure 15:**
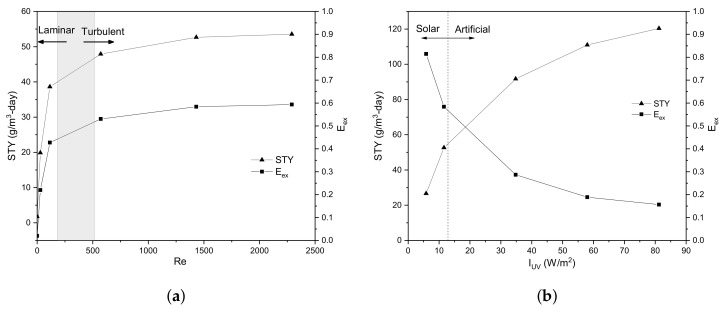
Space–time yield maximisation by (**a**) hydrodynamic regime at a constant superficial radiation intensity (11.6 W/m${}^{2}$) and (**b**) superficial radiation intensity at a constant flow velocity (0.05 m/s).

Figure 15 shows the STY and $E_{ex}$ factors as a function of the Reynolds number (Figure 15a) and superficial intensity radiation (Figure 15b). The STY is the space–time yield factor and relates the amount of dye degraded per unit of time and reactor volume, useful for comparing reactors of any scale. Meanwhile, the $E_{ex}$ is the external effectiveness factor, and serves as an indicator to know if the global degradation reaction is limited by the mass transfer rate (i.e., $E_{ex}\approx 0$) or by the surface kinetics (i.e., $E_{ex}\approx 1$).

In Figure 15a, the grey region represents the hydrodynamic regime transition of the flow. The flow regime is varied by flow velocity in the Reynolds number between 1 and 2300, obtaining a stabilisation of the STY at a Re number of approximately 1500. As the flow rate increases, the residence time decreases and the mass transfer rate becomes greater; consequently, the amount of degraded dye increases and the STY is improved due to the substantial limitation by the mass transfer rate ($E_{ex}$ < 0.5). It is observed that in the laminar regime, there is a significant increase in the STY, compared to the turbulent regime; this is due to the behaviour of the mass transfer rate coefficient in each flow regime. On the other hand, the STY tends to stabilise as the flow becomes turbulent since the mass transfer coefficient does not increase significantly with the flow velocity. In addition, the $E_{ex}$ factor increases for Re to an approximate value of 0.6; that is, the superficial reaction velocity begins to have importance in the overall rate of dye degradation.

Additionally, it is possible to select a flow velocity of 0.05 m/s (equivalent to a Re number of 1431) to maximise the STY at a constant superficial radiation intensity of 11.6 W/m${}^{2}$. The radiation intensity could be increased from this flow rate to improve the STY and intensify the operational variables by understanding the system. If the system is strongly limited by the mass transfer rate, the STY factor can serve as a factor to maximise dye degradation either with flow rate or geometrically. In the case of a limitation due to superficial reaction, the radiation intensity should be increased by an artificial lighting system to increase the STY.

Figure 15b shows the STY as a function of superficial radiation intensity at a constant flow velocity of 0.05 m/s. It is observed that a small change in the radiation intensity generates a significant increase in STY in the solar energy zone, and this is because of a surface reaction limitation ($E_{ex}$ > 0.5). By increasing the superficial radiation intensity greater than 50 W/m${}^{2}$, there is no significant increase in STY due to a mass transfer limitation; therefore, increasing the flow rate at a superficial radiation intensity of 50 W/m${}^{2}$ could be more appropriate.

In this work, the photoanode geometry and the optical thickness (δ) were defined; in addition, a preliminary design of a photoelectrocatalytic reactor was proposed, which consists of a tubular geometry with internal and external illumination capacity. This geometry can be easily scalable by serializing several modules of defined length. It is worth mentioning that other design aspects of the photoelectrocatalytic reactor, such as the module’s geometry (defined by the shape of the inlet and outlet flow), the length, and operating conditions, will be discussed in our following study. The preliminary design of the photoelectrocatalytic reactor proposed in the present work can be used in other applications, for example, in the degradation of other emerging contaminants, such as pharmaceutical residues, pesticides, or other kinds of textile dyes. Some dimensions may change according to the application of the photoreactor; for example, the optical thickness will depend on the absorption coefficient of the medium, which strongly depends on the type of dye or contaminant. It is possible that when using another dye, such as vat, a lower optical thickness is required to reduce radiation losses. However, the procedure to carry out this work can be used to design a photoelectrocatalytic reactor that degrades any pollutant.

## 4. Conclusions

The configuration of a photoelectrocatalytic reactor to degrade Reactive Red 239 (RR239) dye was developed using a numerical approach, based on analysis of mass transfer, momentum, radiation, and surface kinetics.

The position of the photoelectrode inside the reactor was determined based on the optical thickness (δ) or distance between the external surface and the photoelectrode. It was found that both the rate of mass transfer and the loss of radiation intensity show a significant dependence on δ, especially for waters with high dye concentration (>60 mg/L), due to the high losses of radiation intensity in the UV and visible spectrum, being greater in the latter. For the specific case of this work, a RR239 dye concentration of 6 mg/L, a δ of 1 cm is adequate to maximise the mass transfer rate while maintaining low radiation intensity losses (<15%).

A woven mesh geometry was selected for the photoelectrode, which produces higher levels of micromixing in the vicinity of the electrode, and consequently higher mass transfer rates compared to the expanded mesh geometry. It was possible to obtain up to 6 and 3 times the mass transfer rate using a woven mesh photoelectrode compared to the case of photocatalyst deposited on the external surface (without mesh electrode) for the laminar and turbulent regimes, respectively.

The space–time yield (STY) was maximised to a value of approximately 110 g/m${}^{3}$-day by mass transfer rate and reaction kinetic analysis, identifying cases of mass transfer limitation or reaction rate limitation. With this work, a preliminary methodology based on a numerical approach was proposed for the study of photoelectrocatalytic reactor configurations to degrade textile dyes, which can be used with other types of dyes or a mixture of them.

## Appendix A. User Define Functions Pseudocodes

### Appendix A.1. Turbulent Schmidt Number

This is the UDF pseudocode to calculate Turbulent Schmidt number in Fluent solver.

**Algorithm 1:** Turbulent Schmidt number determination.
**Required:** $D_{RR234}$, $Sc_{\infty }$, ρ **Result**: Turbulent Schmidt number calculation into the Fluent solver (**/*** Define this UDF macro to specify the turbulent Schmidt number into the solver"( ***/** DEFINE_TURB_SCHMIDT() Macro Obtain turbulent properties $\mu_{T}$(C_MU_T(c,tc)) Calculate Turbulent Schmidt number using Equation (20) **Return** result

### Appendix A.2. Mass Transfer Coefficient

This is the UDF pseudocode to calculate the mass transfer coefficient in laminar and turbulent regime in Ansys Fluent software.

**Algorithm 2:** Mass transfer coefficient determination.

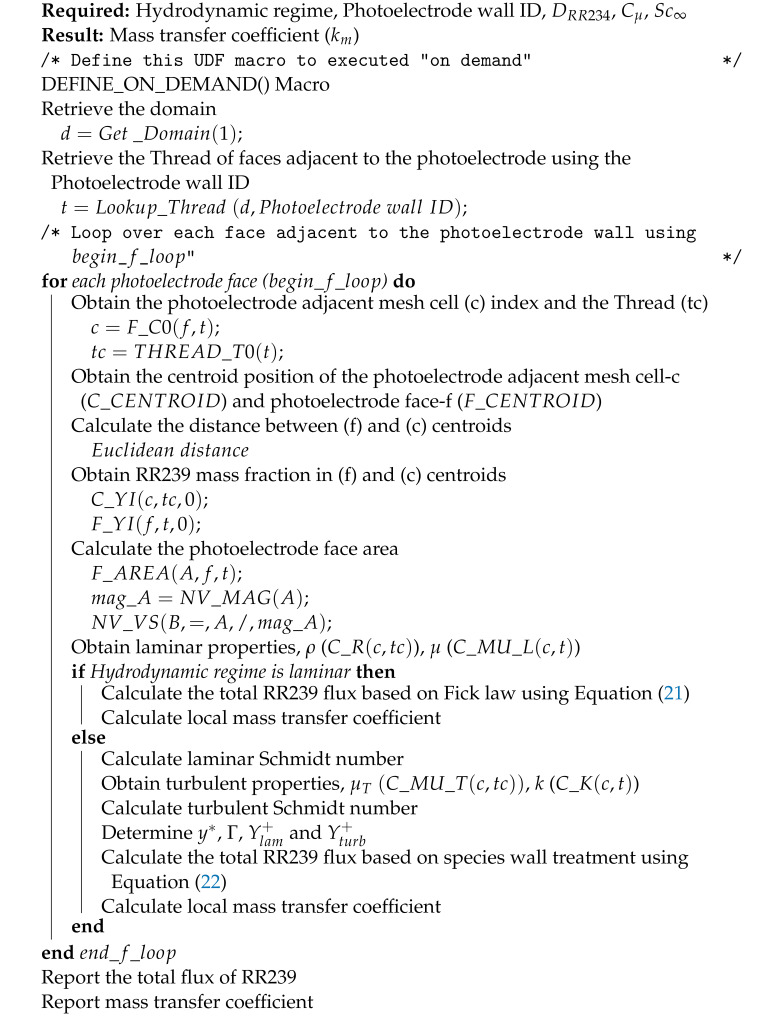

### Appendix A.3. Surface Reaction

This is the UDF pseudocode to define the custom photoelectrocatalytic reaction rate.

**Algorithm 3:** Mass transfer coefficient determination.
**Required:** All kinetic constants parameters, Surface radiation intensity (**/***Create the kinetic reaction function( ***/** realreaction( ) Obtain fluid density ρ (C_R()) Obtain the dye mass fraction (with specie ID) and calculate the molar concentration **Return** reaction equation (Equation (25)) (**/***Define this UDF macro to specify the custom surface reaction rate over the photoelectrode wall( ***/** DEFINE_SR_RATE() Macro Call the reaction function created above and assign it to the reaction term (*rr) in the UDF macro
